# A dislodged coronary stent migrating over a guiding catheter: a “Muckers-type” stent loss retrieved using a snare technique

**DOI:** 10.1186/s43044-025-00703-6

**Published:** 2025-11-16

**Authors:** Michał Kuzemczak, Wojciech Suślik, Michał Stachura

**Affiliations:** 1 Division of Emergency Medicine, Poznan University of Medical Sciences, Poznań, Poland; 2 Department of Cardiology, Biegański Hospital, Catheterization Laboratory, Medical University of Lodz, Łódź, Poland; 3 Department of Interventional Cardiology and Internal Diseases, Military Institute of Medicine - National Research Institute, Legionowo, Poland; 4 Department of Internal Diseases and Cardiology, Medical University of Warsaw, Warsaw, Poland

**Keywords:** Stent dislodgement, Stent loss, Stent retrieval, Snaring

## Abstract

**Background:**

Coronary stent dislodgement is a rare complication of percutaneous coronary interventions with potentially serious clinical sequelae. There are several scenarios for the dislodgement and various techniques to deal with this complication. A dislodged stent migrating retrogradely over a guiding catheter and retrieved with a snare system has never been reported. We present the first reported case of a dislodged coronary stent passing over a guiding catheter which was successfully retrieved with a snare system.

**Case report:**

A 76-year-old patient with unstable angina was admitted for a coronary angiography. It revealed in-stent restenosis in the mid portion of the left anterior descending artery (LAD) and within the ostium of the left main coronary artery (LM). Following an uncomplicated PCI of the LAD and during subsequent stent deployment in the LM, the balloon with a mounted stent popped out to the aorta. After the balloon had been pulled back, the stent migrated over the guiding catheter. Consequently, another longer stent was successfully implanted into the LM, and afterwards the dislodged stent was snared and retrieved using an ipsilateral femoral access. The patient was discharged after two days of uneventful hospital stay.

**Conclusions:**

The case report demonstrates an unusual course of stent dislodgement passing over a guiding catheter which was successfully retrieved with a snare technique. It underscores the importance of optimal lesion preparation and meticulous vigilance when implanting short stents into LM.

## Introduction

Coronary stent dislodgement is a rare complication of percutaneous coronary interventions (PCI) with potentially serious clinical sequelae such as coronary occlusion, systemic or coronary embolization, emergency surgery, bleeding complications, and in the worst case scenario, death [1]. Although the incidence of coronary stent dislodgement has declined with the advent of more advanced coronary stents and expanding array of treatment modalities developed for adequate lesion preparation, this complication may still occur given the increasing complexity of patients undergoing PCI [2–6]. Severely calcified lesions, ostial lesions, diffuse lesions, pronounced vessel tortuosity as well as coronary arteries with previously implanted stents are well-documented factors associated with a higher risk of stent loss [7, 8]. Therefore, PCI operators should be aware of such a complication and, most importantly, be familiar with techniques to effectively deal with this potentially fatal procedural event.

There are several clinical scenarios of stent dislodgement and, accordingly, a plethora of techniques and devices have been developed to manage this PCI-related complication (e.g. snares, basket retrieval devices, various types of forceps). Historically, the incidence of coronary stent dislodgment has been estimated to be around 1.4–3.4% and has decreased to 0.07–0.58% in the modern drug-eluting stents (DES) era, but a dislodged stent migrating retrogradely over a guiding catheter and successfully retrieved with a snare system has never been reported [8]. Herein, we present such an exceptionally rare case which was successfully managed with the use of a snare technique via ipsilateral femoral approach.

## Case report

A 74-year-old male patient with a history of chronic coronary syndrome, hypertension, dyslipidemia and peripheral artery disease was admitted due to unstable angina symptoms. Prior to the present admission the patient had undergone several coronary interventions, i.e. PCI of the right coronary artery (RCA) with a bare metal stent (BMS) implantation– sixteen years prior; PCI of the left anterior descending artery (LAD) with two BMSs implantation in the course of ST-elevation myocardial infarction (STEMI) – fifteen years prior; PCI of the LAD with a drug-eluting stent (DES) implantation – seven years prior, and PCI of the LM/LAD with DES implantation – one year prior.

**Fig. 1:**
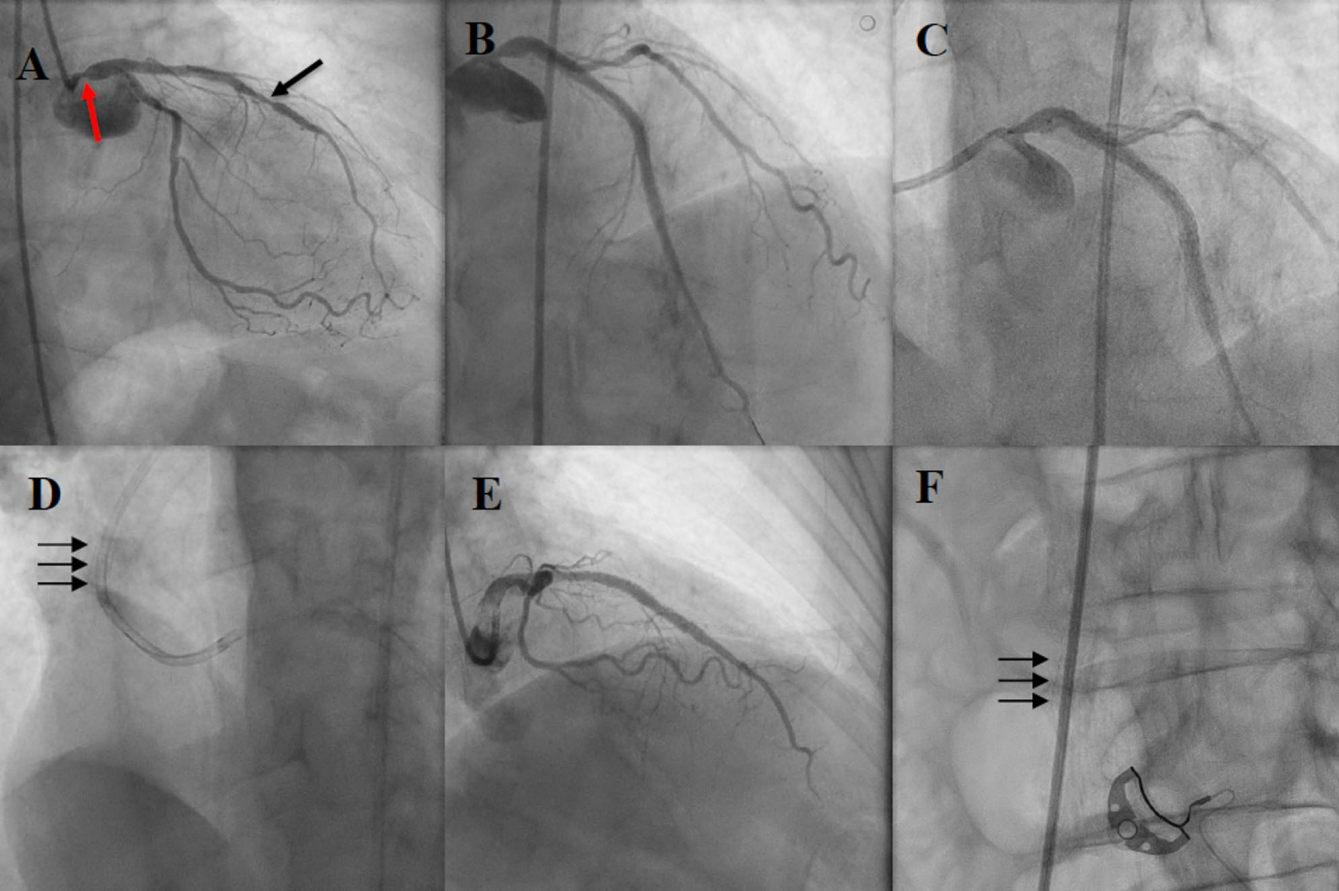
PCI of the mid LAD and ostial LM resulting in stent loss. **A** Left coronary angiography demonstrating in-stent restenosis in the midLAD (black arrow) and ostial LM (red arrow); **B** Left coronary angiography following DES implantation in the midLAD; **C** Positioning of DES in the ostium of the LM; **D** The dislodged stent migrated retrogradely and encircled the guiding catheter (arrows); **E** Left coronary angiography following DES implantation into the ostium of the LM; **F** The dislodged stent migrated with the blood flow to the right common iliac artery (arrows)

The present procedure was performed via right femoral approach since both radial arteries were occluded. Coronary angiography revealed a 75–80% narrowing in the mid LAD (in-stent restenosis) and 70–80% ostial lesion in the LM (edge in-stent restenosis within a 5 mm segment adjacent to the previously implanted stent) (Fig. 1A). Intravascular ultrasound (IVUS)-derived minimal lumen area (MLA) in the ostium of the LM was 4.4 mm^2^.

During the procedure, a 6-French Judkins left guiding catheter (Launcher, Medtronic, Minneapolis, MN, US) was used and a Sion Blue coronary guidewire (Asahi Intecc Medical, Irvine, CA, US) was passed to the distal portion of the LAD. Following successful implantation of an Ultimaster Nagomi 3.5/28.0 mm DES (Terumo Corporation, Tokyo, Japan) into the mid LAD (Fig. 1B), ostial disease in the LM was addressed. The lesion was pre-dilated with a semi-compliant Artimes 3.5/15.0 mm balloon (Brosmed, Amersfoort, Netherlands) (16 atm) and, subsequently, an attempt was made to implant a Xience 4.0/8.0 mm DES (Abbott Vascular, Santa Clara, CA, US) (Fig. 1C). However, during subsequent inflation of the stent balloon in the left main, it slipped from the desired location to the ascending aorta resulting in inadvertent expansion of the stent outside the coronary tree. After the balloon had been pulled back, the stent migrated retrogradely over the guiding catheter. It was found outside the patient’s coronary tree encircling the guiding catheter above its secondary curve (Fig. 1D). The decision was made to stent the ostium of the LM first and, afterwards, to deal with the dislodged stent. Therefore, an Ultimaster Nagomi 4.0/12.0 mm DES (Terumo Corporation, Tokyo, Japan) was implanted into the LM and post-dilated with a non-compliant Aperi 5.0/10.0 mm balloon (Brosmed, Amersfoort, Netherlands) (20 atm) (Fig. 1E). Post-procedural IVUS-derived MLA 14 mm2 was achieved.

**Fig. 2:**
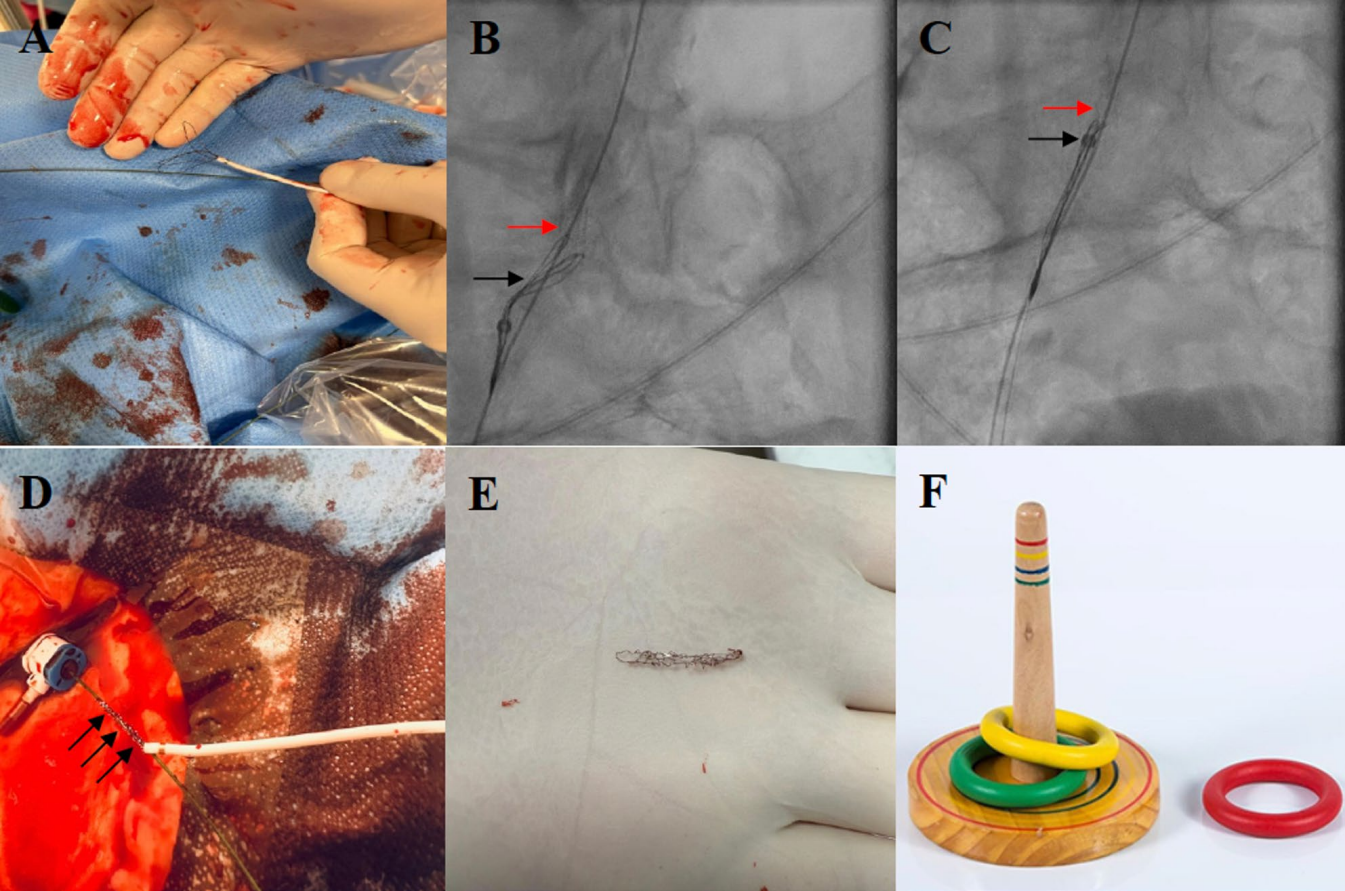
Retrieval of the dislodged stent with a snare system. **A** One of the loop of the multi-loop snare was passed through the end of the 0.035” wire; **B** The multi-loop snare (black arrow) was advanced with a delivery catheter adjacently to the dislodged stent in order to capture the stent (red arrow); **C** The captured stent (red arrow) was pulled back towards the delivery catheter (tip of the delivery catheter – black arrow); **D** The dislodged stent (black arrows) encircling the 0.035” wire successfully retrieved from the patient’s vasculature; **E** The dislodged stent retrieved out of the patient’s vasculature; **F** Muckers - a traditional game, in which players throw circular rings at a stick

When PCI of the LM was performed, the dislodged stent migrated with the blood flow along the guiding catheter to the right common iliac artery (Fig. 1F). First, in order to retrieve the dislodged stent, the coronary guidewire and the balloon were withdrawn. Subsequently, a 0.035” guidewire was introduced into the guiding catheter. Then, the guiding catheter was gently pulled back leaving the stent on the guidewire and a 6-French femoral sheath was exchanged into a 10-F sheath. Subsequently, a multi-loop snare Atrieve 18.0/30.0 mm (Argon Medical, Frisco, TX, US) was passed through the end of the 0.035” wire (Fig. 2A) and advanced with a delivery catheter adjacently to the dislodged stent (Fig. 2B). The stent was captured (Fig. 2B) and pulled back towards the delivery catheter (Fig. 2C). Once resistance during traction was encountered, it was decided to remove the entire system including the 0.035” guidewire, the stent and the multi-loop snare along with its delivery catheter (Fig. 2D). *En bloc* retrieval resulted in successful capture of the dislodged stent, which ex vivo demonstrated a severe structural distortion (Fig. 2E). Finally, the procedure was completed by closing a 10-French femoral access site with an 8-French AngioSeal (Terumo Corporation, Tokyo, Japan) vascular closure device. The postprocedural period was uneventful and the patient was discharged two days following the procedure.

## Discussion

Stent dislodgement is infrequent, but potentially a life-threatening complication. According to different studies, in contemporary PCI era its incidence is estimated to range from 0.29% to 0.32% [1]. Importantly, the incidence of stent loss has significantly decreased over the years, since contemporary coronary stents are mounted onto balloons in a contracted condition by manufacturers (pre-mounted stents) compared to their older predecessors which had to be manually crimped before implantation (hand-mounted stents) [2, 3]. This does not mean that stent dislodgement has been totally eliminated from everyday clinical practice. Furthermore, this procedure-related event has the potential to cause fatal complications. Therefore, PCI operators should still be aware of such a risk and be familiar with different retrieval strategies.

Interestingly, results derived from bench tests indicate that various contemporary stents differ when it comes to the risk of being peeled off from their mounting balloons. Namely, in the study by Seo et al., Orsiro stent was more susceptible to dislodgement compared to other counterparts (i.e. Xience Sierra, Firehawk, Resolute Onyx, Synergy) evaluated in the bench tests [8]. This implies that one should bear in mind different stent designs and their characteristics when treating particular lesions. In the present case, a Xience stent was used which is one of the most widely studied stents worldwide with very good performance and low complication rates. Despite that, the stent loss occurred which underscores the importance of constant vigilance when PCI is performed, even with devices exhibiting an exceptional safety profile.

Several factors predisposing to the above-mentioned complication have been described and they include inadequate lesion preparation, heavily calcified plaques, ostial lesions, vessel tortuosity, previously stented segments and non-coaxial position of a guiding catheter [1, 7, 8]. In the present case, one of the targets for PCI was ostial lesion in the LM which had been previously partially covered with a stent. Furthermore, a relatively short stent was primarily selected, which could have been more prone to popping out of the LM ostium than longer stents. Therefore, the second-choice stent was longer, providing better stability within the lesion and its implantation was successful. Furthermore, in such cases, slow stent inflation and disengagement of a guiding catheter from the LM ostium can make the stent implantation more controllable. Lastly, sometimes it is worth to ask a patient to minimize respiratory movements or stop breathing for a moment since this can make the stent implantation more predictable.

The unique aspect of this case is that the dislodged stent migrated over the guiding catheter. The stent’s diameter following balloon inflation was large enough to easily pass over the 6-French guiding catheter. Namely, the outer diameter of the latter is ca. 2.0 mm, while the stent’s diameter following inflation was at least 4.0 mm. Therefore, during withdrawal of the balloon into the guiding catheter, the stent encircled the guiding catheter and traveled with the blood stream to the patient’s right common iliac artery. Due to its resemblance to a ring over a stick in the traditional outdoor Muckers game (Fig. 2F), we propose the term *“Muckers-type stent loss”* to describe this specific and exceptionally rare mechanism of stent dislodgement.

There are several retrieval techniques and devices that have been developed for intravascular foreign body retrieval. Some of them utilize devices which have been specifically designed to remove foreign bodies from patients’ vasculature (e.g. single- and multi-loop snares, baskets, various forceps), while other techniques exploit regular armamentarium from the interventional cardiologist’s toolbox (e.g. small balloon technique, twisted guidewire technique, “hairpin-trap” technique, self-made snares) [1, 9, 10]. One of the key aspects of dealing with a dislodged stent is keeping a wire in situ at all times – this makes stent retrieval easier. At the worst case scenario, when retrieval attempts deem futile, a dislodged stent can be crushed by another stent (preferably in a peripheral vascular bed). However, in our opinion, a dislodged stent should not be left in a patient’s vascular system as long as there is a possibility of removing it. To the best of our knowledge, this is the first reported case in the available literature illustrating such a unique example of stent dislodgement managed successfully with a snare technique. The latter is often the preferred retrieval strategy due to its efficacy and safety profile, but the technique has never been used in such a scenario. Mondal et al. reported the only case of a dislodged stent passing over a guiding catheter, but in order to avoid surgical retrieval, it was managed by deployment of the stent in the patient’s external iliac artery [11]. Contrary to our findings, the authors suggested that percutaneous retrieval of fully deployed stents should not be recommended. Alternatively, if the stent cannot be removed through the sheath, it may be crushed against the iliac or femoral artery with a peripheral stent [12]. Lastly, if a dislodged stent is located in a small peripheral artery branch (e.g. femoral artery branch), the risk associated with its retrieval may outweigh the risk of local complications [1]. In such instances, it may be better to leave the stent in place and maintain clinical surveillance [1].

Of note, in this particular case, a 6-French femoral sheath was upsized to a 10-French sheath. Upsizing of the vascular sheath was made in order to reduce the risk of the stent getting stuck at the tip of the femoral sheath being too small to accommodate the distorted stent, as described previously by Brilakis et al. [1]. Namely, it was anticipated that the snared stent might be deformed and elongated, therefore, a larger lumen of the sheath would make the procedure less cumbersome and would reduce the risk of leaving any stent remnants in peripheral vasculature. Ultimately, the femoral access site was closed with an 8-French AngioSeal (Terumo Corporation, Tokyo, Japan) vascular closure device. This approach, albeit off-label, has been demonstrated safe and effective for arteriotomies ranging from 9-French to 12-French [13].

## Conclusions

The presented case report demonstrates an unusual scenario of stent loss posing a unique interventional challenge. In such cases, prompt recognition and proper management are essential in order to avoid complications and their sequela, including fatal cardiovascular events. Despite its rarity, PCI operators should be aware of such a course of stent dislodgment and be familiar with available retrieval techniques ensuring patient safety and good clinical outcomes. In the present case, the snare method applied was effective, highlighting its utility in such a unique scenario.

## Data Availability

No datasets were generated or analysed during the current study.

## Abbreviations

PCI: Percutaneous coronary intervention
LM: Left main coronary artery
LAD: Left anterior descending artery
RCA: Right coronary artery
MLA: Minimal lumen area
IVUS: Intravascular ultrasound
ISR: In-stent restenosis
